# A paper-based competitive lateral flow immunoassay for multi β-agonist residues by using a single monoclonal antibody labelled with red fluorescent nanoparticles

**DOI:** 10.1007/s00604-018-2730-9

**Published:** 2018-02-22

**Authors:** Ruiguo Wang, Wei Zhang, Peilong Wang, Xiaoou Su

**Affiliations:** 1 Key Laboratory of Agro-product Safety and Quality, Ministry of Agriculture, Beijing, 100081 People’s Republic of China; 2 Institute of Quality Standards & Testing Technology for Agro-Products, Chinese Academy of Agricultural Sciences, Beijing, 100081 People’s Republic of China

**Keywords:** β-Agonist, Fluorescent nanoparticles, Paper-based assay, Immunoassay, Multi residues, Pork tissue

## Abstract

An ultrasensitive paper based lateral flow assay is described for rapid and simultaneous fluorometric detection of several β-agonists including clenbuterol and its chemical analogues (mabuterol, brombuterol, cimaterol, cimbuterol, bromchlorbuterol and banbuterol). A nonspecific monoclonal antibody (mAb) against clenbuterol and its analogues was prepared and employed in a competitive immunoassay where mAb conjugated to fluorescent nanoparticles and free β-agonists compete for the binding sites. This enables rapid screening for the 7 β-agonists in a single run that takes about 8 min. Detection limits for the seven β-agonists are <50 pg g^−1^ of pork. Recoveries ranged from 69.5% to 102.4%, and relative standard deviations were ±15%. The assay was applied to the analysis of both using spiked and unspiked pork for β-agonists, and the results compare well to those obtained by HPLC-MS.

**Electronic supplementary material:**

The online version of this article (10.1007/s00604-018-2730-9) contains supplementary material, which is available to authorized users.

**Graphical abstract:**
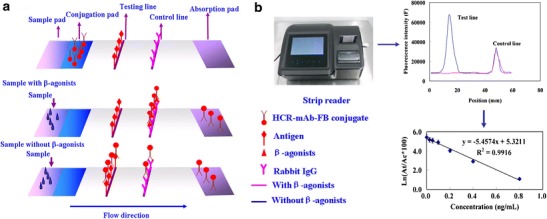
Schematic presentation of an ultra sensitive fluorescent nanoparticle based paper based assay for rapid detection of multi β-agonists in pork tissue.

## Introduction

β-Agonists are a family of synthetic phenylethanolamine compounds used for treatment of human pulmonary disease and asthma. They can be divided into 3 groups with different aromatic core structure: aniline, phenol and resorcinol [1]. β-Agonists promote the growth and nutrient repartition for swine, cow and other livestock [2]. Residues of β-agonists carried over by feed or drinking water can lead to hazard effects on human health [3]. Hence, β-agonists have been banned as growth promoters in the European Union [4] and China [5]. Therefore, sensitive, broad screening and convenient assays are needed for onsite detection of trace residues of β-agonists.

Several analytical techniques based on expensive instrument, such as liquid chromatography (LC) [6, 7], gas chromatography-mass spectrometry (GC-MS) [8], LC-tandem mass spectrometry (LC-MS/MS) [9], GC-MS/MS [1] and capillary electrophoresis [10], have been applied into detect trace amount of β-agonist residues in various samples. Above methods are complicated and laborious because they involve time-consuming sample preparation and chromatographic separation procedures. Some novel sample preparation such as molecularly imprinted polymer was used [11] to improve clean-up, but the procedure was still time consuming. Enzyme immunoassay [12] and radio-immunoassay [13] didn’t require expensive instruments, but the incubation and washing procedures were complicated. Currently, some novel analytical method, such as electrochemical sensors [14], micro fluidic chips [15], surface-enhanced Raman scattering immunoassay [16], sensitive visual probes [17, 18], and dual-responsive fluorescence [19] have been developed. Cao et al. [20] have developed the novel immuno sensor for clenbuterol (CLEN) by coupling purification and in situ immobilization process of monoclonal antibodies. However, these newly developed methods were subjected to either single component detection or lower sensitivity, which don’t meet the demand of emerging requirements of broad screening and onsite detection.

Paper has become a simple, flexible, and reliable platform for analytical devices. One of the most familiar applications of paper based analytical devices is a lateral flow immuno assay (LFIA), which is broadly used into detection of small molecules and biomarkers [21]. Compared with conventional detection methods, LFIA offers advantages such as low cost, mutable fabrication and speed. The most usually used format of LFIA is the employment of colloidal gold as reporters for colorimetric detection, which can realize qualitative or semi quantitative analysis of the target analytes. However, such format of LFIA can only be used for analyzing target analytes with relatively high concentrations. Colloidal gold based LFIA has been applied to the qualitative detection of CLEN and salbutamol (SAL) [22, 23]. In order to improve sensitivity of LFIA for β-agonists, labels such as Ru(phen)_3_^2+^ doped silica nanoparticle [24] and fluorescent nano silica [25], have been employed in LFIA fabrication. In 2015, we have developed a fluorescent beads based multi component LFIA method for simultaneous detection of CLEN, ractopamine (RAC) and SAL in feed, urine and tissue samples with higher sensitivity (as low as 0.1 ng mL^−1^) [26]. It provided a promising fabrication strategy of multi-component LFIA method. However, the task is daunting as there are many kinds of β-agonist drugs existing in various types of sample matrices such as animal feed, tissue and body fluids. Most of the published LFIA methods for β-agonist detection were limited to no more than three analytes due to capacity of the testing strip and limited degree of cross-reactivity of the antibodies.

In this study, we report a one-step fluorescent lateral flow immunoassay (FLFIA) which can realize screening or semi quantification of CLEN and its structural analogues (See Electronic Supplementary Material Fig. S1), including mabuterol (MAB), brombuterol (BBT), cimaterol (CMT), cimbuterol (CBT), bromchlorbuterol (BCT) and banbuterol (BAN). The method utilizes a high performance antibody with high cross reactivity that can detect trace amounts of seven kinds of β-agonist residue in pork tissue samples. Limits of detection (LODs) as low as 0.05 ng·mL^−1^ (or ng·g^−1^) can be achieved. With the FLFIA assay, ultra sensitive, large scale and high speed screening of illicit β-agonist can be realized in the field with a low cost comparable to colloid gold testing strips currently in practice.

## Experimental

### Regents and apparatus

CLEN, MAB, BBT, CMT, CBT, BCT, BAN, RAC, SAL, fenoterol, tulobuterol, epinephrine, dopamine and other compounds such as penicillin, ampicillin, kanamycin and ciprofloxacin were purchased from Sigma-Aldrich (St. Louis, USA. https://www.sigmaaldrich.com/). The high cross reactive monoclonal antibody (HCR-mAb) of β-agonist (13 mg mL^−1^) and the corresponding antigen (CLEN-BSA, the valence of antigen was about 5000) were jointly developed by our laboratory and Beijing Kingbown Bio-tech LTD (http://www.kwinbon.com/). The rabbit anti-mouse immunoglobulin (IgG) was also from Beijing Kingbown Bio-tech LTD (http://www.kwinbon.com/). Fluorescent carboxy modified latex nanoparticles (F8810, particle size 200 nm, excitation best at 580 nm; enission peak at 605 nm) were purchased from Life Technologies, USA (https://www.thermofisher.com/cn/zh/home/brands/life-technologies.html).

Water was obtained from a Milli Q purification system (Millipore, http://www.merckmillipore.com/). All other chemicals were of analytical grade or better. Nitrocellulose (NC) membranes (CN95, pore size is 15 μm) were from Sartorius (Göttingen, Germany. https://www.sartorius.com.cn). Glass fibers and absorbent pads were obtained from Millipore Corporation (Bedford, USA. http://www.merckmillipore.com/). ZX 1010 Dispensing Platform, LM8030 Batch Laminator and CM4000 Guillotine Cutter from BioDot (Irvine, USA. https://www.biodot.com/) were used to prepare test strips. Fluorescence was detected by a special fluorescence photometer (BT-211D, 30 cm×20 cm×12 cm) provided by Beijing Zhifeng Botai Bio-tech Ltd.

### Preparation of the fluorescent lateral flow immunoassay (FLFIA)

**Fig. 1:**
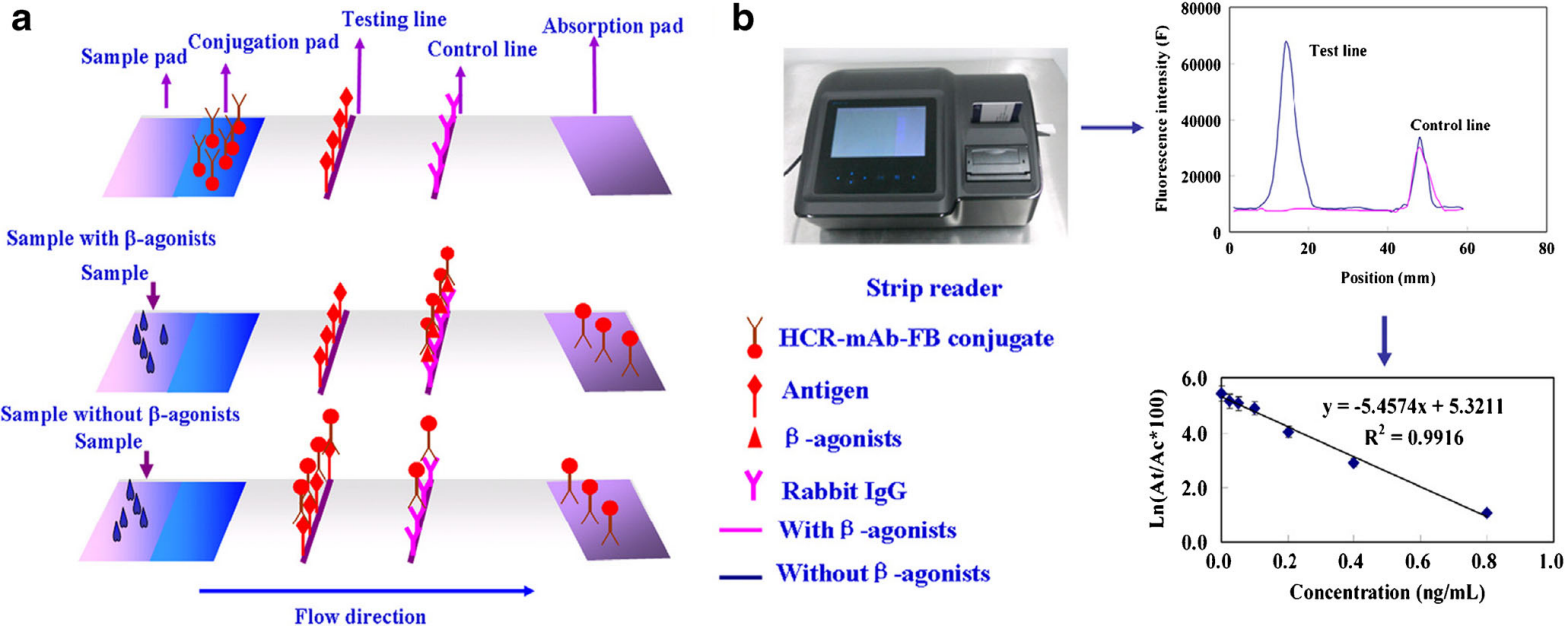
**a** Schematic of the fluorescent lateral flow immunoassay (FLFIA) and the immunoassay procedure for samples. **b** Typical results and calibration curve with FLFIA strip reader

Structure of the test stripe is shown in Fig. 1a. Conjugate pads and sample pads were made from glass fiber. They were treated with blocking buffer (0.01 M phosphate buffer (pH = 7.4) containing 2% OVA, 2% sucrose and 0.02% NaN_3_) and dried at 37 °C over night. The HCR-mAb-FB conjugate was dispensed onto the conjugate pad with the BioDot jet printer and dried. The antigen CLEN-BSA (1 mg/mL) and rabbit IgG were coated on NC membrane as testing line and control line by using a BioDot jet printer with proper rate. After drying at 37 °C for 1 h, the NC membranes were stored in a desiccator. The NC membrane, conjugate pad, sample pad and absorbent pad were laminated and glued to a plastic backing. Then, the assembled pads were cut into strips (0.4 cm × 6.0 cm), which were installed in dip stick shell and stored in desiccator at room temperature until use.

### Immunoassay procedure

For the immunoassay, about 60 μL of β-agonist standard or extracted sample solution was loaded onto the sample pad of the stripe. With the aid of the absorbent pad, the liquid moved along the NC membrane. β-agonists in the sample reacted with HCR-mAb-FB conjugates in the conjugate zone. When there were no β-agonists in the sample, all HCR-mAb-FBs reach the test line and can be captured by the immobilized antigen, which resulted in fluorescence at the highest intensity (Fig. 1a). If there were β-agonists in the sample solution, the HCR-mAb-FBs were occupied by their corresponding β-agonists, and cannot be captured by the immobilized antigen, which resulted in reduced fluorescence intensity. After reaction, fluorescence from the immobilized HCR-mAb-FBs can be detected by a scanning fluorescence photometer and a calibration curve can thus be obtained (Fig. 1b).

### Sample pretreatment

For pretreatment of pork tissue samples, 1 g ground pork was weighed and mixed with 2 mL phosphate buffer in a 10 mL plastic centrifuge tube. After homogenate with homogenizer (IKA, Germany), the tube was centrifuged for 5 min at 10000 rpm and the supernatant was collected for FLFIA analysis.

### Fluorescence detection

Fluorescence of immobilized FB-mAbs on test line and control line of the strip was detected by a scanning fluorescence photometer. The strip was inserted into sample holder of the fluorescence photometer and the scan head of the photometer moves along the reading zone of the FLFIA strip, where the test lines and control line were scanned in sequence. The fluorescent signals of test line and control line was obtained for calculating detection results.

### Method validation

The validity of the method was verified by analyzing pork tissue samples individually spiked with seven kinds of β-agonist at different levels. The results of real contaminated pork tissue samples which were from China National Feed Quality Control Center (Beijing), were used to further validate and compare with results from ultra performance liquid chromatography coupled tandem mass spectrometry.

## Results and discussion

### Evaluation of HCR-mAb for β-agonists

Antibody plays a crucial role in the method. In this study, the production method of the HCR-mAb was similar with the published method [27]. Briefly, CLEN was linked to BSA and then used as immunogen. The immunogen of CLEN-BSA was injected into Balb/c mice and each mouse was given 150 μg of immunogen subcutaneously in Freund’s complete adjuvant to produce antisera. After the fusion and hybrid selection of the spleen cells from mice and SP2/0 bone marrow tumor cells at proper ratio (8:1) to achieve the hybrid tumor cell line of monoclonal antibody. For the production and purification of HCR-mAb, the sterilization paraffin oil was injected into abdominal cavity of the Balb/c mice. After 7 days, each mouse was given 4×10^5^ of recovery hybrid tumor cell line. The ascitic fluid from the mice was accumulated after 7 days and then was purified to achieve the mAb against aniline β-agonists. Furthermore, the performance of the novel HCR-mAb of the immunoassay such as sensitivity and specificity was examined in detail. The results of half maximal inhibitory concentration (IC50) and cross reactivity (CR) were listed in Table S1. The results indicated that the HCR-mAb has excellent sensitivity and specificity towards aniline β-agonists such as CLEN, MAB, BAN, BBT, CMT, CBT and BCT. Especially for CMT, CBT and BCT, IC50 values as low as 0.09 ng·mL^−1^ can be achieved. The novel HCR-mAb showed much lower sensitivity towards phenol and resorcinol β-agonists such as RAC, SAL, fenoterol, tulobuterol and other veterinary drugs. Typical IC50 values for concentrations of competitor drug were above 100 ng·mL^−1^. In addition, cross reaction experiments showed that the HCR-mAb can only recognize β-agonists containing aniline group with high specificity. For the antibiotic compounds, the CR of the developed HCR-mAb was lower than 0.013%. For other β-agonists containing phenol and resorcinol such as RAC, SAL, fenoterol and tulobuterol, the CR was only 0.13%. Even for the natural compounds such as epinephrine and dopamine, the CR was below 0.065%. The CR results indicated that the HCR mAb possessed satisfactory specificity for aniline group β-agonists, it enabled high through screening for aniline group β-agonists even though with presence of natural compounds like β-agonist such as epinephrine and dopamine. For the cross reactivity improvement of HCR-mAb, it was speculated that the active site of the novel HCR-mAb was specific for aniline β-agonist due to the active site on the HCR-mAb can selectively bind aniline on the benzene ring of β-agonist.

### Optimization of HCR-mAb-FB conjugate preparation

In accordance with our previous study [26], carboxylate modified FB was first activated in 0.01 M MEST buffer (pH = 5.0) for 10 min, then reacted with certain concentration of HCR-mAb in phosphate buffer (0.01 mol L^−1^). The optimal concentration of HCR-mAb for FBs labeling was obtained using “trial and error” method. HCR-mAb solution at concentrations of 1.0 mg mL^−1^, 2.0 mg mL^−1^ and 4.0 mg mL^−1^ were examined and it was found that sensitivity of the detection system was highest at 2.0 mg mL^−1^ and the volume of the HCR-mAb solution was 100 μL. In addition, fluorescence of the FBs did not show any shift after surface modification, the excitation wavelength was still 580 nm and the emission wavelength was 605 nm.

### Optimization of the fabrication of the strip for the lateral flow immuno assay

In the previous study [26], we optimized crucial parameters of strip, such as blocking buffer and NC membrane, to achieve best sensitivity and specificity.

Here, the amount of HCR-mAb-FB and CLEN-BSA antigen was focused and optimized. The amount of HCR-mAb-FB loaded on the glass fiber can seriously influence the final fluorescence intensity at the test and control line on the strip. Stock HCR-mAb-FB solution was diluted by 10-fold, 50-fold and 80 fold. The 50-fold dilution, which accounts of a HCR-mAb-FB surface coverage of 70 μL cm^−2^ was found to provide the best sensitivity with low interference fluorescence signals. The amount of CLEN-BSA antigen was evaluated with “checker-board titration” in ELISA. Finally, the optimal amounts for antigen of CLEN-BSA used for the test lines were 1000 ng cm^−1^.

### Evaluation of the method

#### Reaction time

For the HCR-mAb-FB, it takes time to react and diffuse through the NC membrane after loading sample solution to reach the test line and the control line in the reading zone. The reaction time was optimized using the blank phosphate buffer. The test strip was placed into the photometer and the fluorescence signal from the test line and control line was got at various time intervals. The fluorescence signals of the HCR-mAb-FB on test line and control line reached plateau after 6 min of incubation (Fig. S2). In order to ensure completeness of the reaction and reproducibility of the data, 8 min was selected as the final reaction time.

#### Analytical performance of the strip

**Fig. 2:**
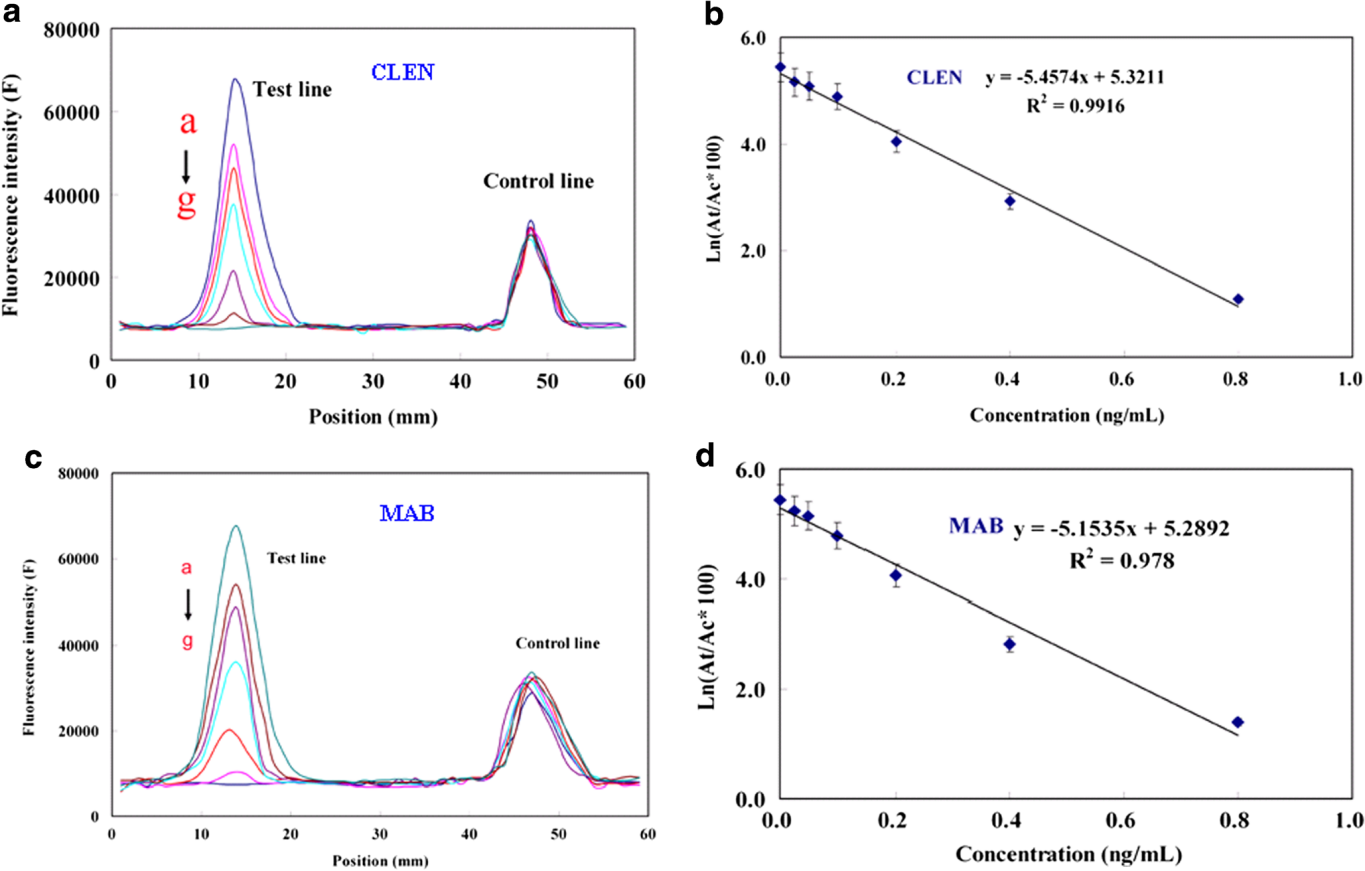
**a** Fluorescence intensity corresponding to 0 ng/mL, 0.025 ng/mL, 0.05 ng/mL, 0.1 ng/mL, 0.2 ng/mL, 0.4 ng/mL and 0.8 ng/mL of CLEN (curves a-g, Ex 580 nm and Em 605 nm) (**b**) Calibration curve of CLEN. **c** Fluorescence intensity corresponding to 0 ng/mL, 0.025 ng/mL, 0.05 ng/mL, 0.1 ng/mL, 0.2 ng/mL, 0.4 ng/mL and 0.8 ng/mL of MAB (curves a-g, Ex 580 nm and Em 605 nm) (**d**) Calibration curve of MAB

As a competitive assay, the fluorescence intensity measured by the photometer decreases with increasing target β-agonist concentration and a logarithm relationship is observed. The fluorescence signal under different concentrations of CLEN and MAB from 0.0 ng·mL^−1^ to 0.8 ng·mL^−1^ was obtained (Fig. 2a, c), the correlation coefficient (R^2^) for CLEN and MAB was 0.9916 and 0.978, respectively (Fig. 2b, d). Five other target β-agonists showed similar behavior (See Table S2).

LODs for the 7 β-agonists are estimated to be lower than 0.05 ng·mL^−1^ (Table S2). Especially for CMT, an LOD as low as 0.01 ng·mL^−1^ is achieved. The limits of quantification (LOQs) for screening of all target aniline β-agonists are estimated to be 0.1 ng·mL^−1^ (ng g^−1^), in which took the dilution fold of sample preparation into account. The LODs of the high throughput strip assay are far better than that of the colloidal gold or fluorescent-based strip method and the LOQs of the FLFIA method can meet the strict minimum residue of limited (MRL) requirement of 0.1 ng/mL for EU.

#### Interferences tolerance for sample matrix

In order to achieve sensitive and specific detection of real samples, it is essential to evaluate interference tolerance of the broad-screening FLFIA method for sample matrix. Animal tissue (pork, veal and steak) were collected to validate specificity of the FLFIA. The results showed that the sample matrices of edible animal tissue had minimum interference on the FLFIA strip after dilution. Due to high sensitivity of the assay, after 2 fold dilution, the assay can still meet or exceed LOD requirement of most standards currently in use in China and EU.

#### Accuracy of the method

Accuracy of the fluorometric lateral flow immunoassay for broad screening was tested with pork tissue samples spiked with target β-agonists. 1 g of tissue spiked with CLEN, MAB, BAN, BBT, CMT, CBT and BCT ranging from 0.2 ng to 1.0 ng. The results are listed in Table 1. Recoveries of CLEN, MAB, BAN, BBT, CMT, CBT and BCT in pork tissue samples are ranged from 69.5% to 102.4%, and the relative standard deviations (RSDs) are below 12%, thus, CLEN, MAB, BAN, BBT, CMT, CBT and BCT in pork tissue can be quantitative determined, and the accuracy and repeatability is satisfactory.

**Table 1:** Recovery of β-agonist individually added to pork tissue samples by using the fluorometric lateral flow immunoassay (FLFIA) method. (*n* = 6)

Analytes	Pork tissue (ng/g)		
	Added	Found	Recovery (%)
CLEN	0.2	0.174	87.0±8.2
	0.4	0.345	86.3±6.5
	1.0	1.024	102.4±4.3
MAB	0.2	0.165	83.5±9.7
	0.4	0.326	81.5±6.6
	1.0	0.896	89.6±5.8
BAN	0.2	0.139	69.5±8.0
	0.4	0.315	78.8±5.1
	1.0	0.857	85.7±4.7
BBT	0.2	0.138	69.0±7.2
	0.4	0.358	89.5±5.0
	1.0	0.938	93.8±6.6
BCT	0.2	0.153	76.5±8.8
	0.4	0.383	95.8±4.9
	1.0	0.927	92.7±5.2
CMT	0.2	0.167	83.5±5.9
	0.4	0.319	79.8±3.8
	1.0	0.874	87.4±2.9
CBT	0.2	0.179	89.5±8.6
	0.4	0.348	87.0±3.3
	1.0	0.952	95.2±4.3

Performance of the method for the detection of β-agonists in the presence of other β-agonists (eg. RAC, SAL) in swine urine was also evaluated. A 1 mL sample of blank swine urine was spiked with 0.1 ng of CLEN, CMT, MAB, CLEN/CMT and CLEN/CMT/MAB, respectively. RAC and SAL were also added into the spiked urine samples. The concentrations of RAC and SAL were 1.0 ng/mL, respectively. The presence of RAC and SAL show no interference on fluorescence signal of CLEN, CMT, MAB, CLEN/CMT and CLEN/CMT/MAB (Fig. S3) and the recovery of CLEN, CMT and MAB in pork tissue samples are from 87.3% to 92.7%. The results are also cross validated with LC-MS/MS. Other possible compounds such as antibiotic including penicillin, ampicillin, kanamycin and ciprofloxacin etc. showed no interference, too. It is noted that the sample spiked with mixed target β-agonists, such as CLEN/CMT and CLEN/CMT/MAB only qualitative determination can be achieved. However, if with the presence of mixed target β-agonists, the fluorescent signal of the FLFIA can significantly reduce with the increase of the types of target β-agonists. Therefore, it is demonstrated that the FLFIA method can perform broad screening of target β-agonists in real samples. Further, the accurate amount of each compound should be determined by LC-MS/MS.

#### Stability of the method

For stability test, tests strip from the same batch were tested during a 3-month period. The strips were stored at room temperature. 10 strips were selected each month and tested with phosphate buffer spiked with 0.2 ng·mL^−1^ of CLEN. The FLFIA strip shows no degradation in performance during 3 months of storage, RSD of the test results for CLEN is 9.3%. Thus, the FLFIA strips show good stability in the long-term test.

### Comparison of analytical performance with published methods

A series of immunoassay methods such as ELISA [12], electrochemical sensors [14], time-resolved chemiluminescence [28] and LFIA [29] have been reported for multi-residue analysis of β-agonists. LFIA method is very convenient and more suitable for field screening. Most of the reported LFIAs for β-agonist can analyze only one target analyte [22, 25]. Some multi-component LFIAs have been developed, which can analyze two or three kinds of β-agonist simultaneously [26, 28]. But none of the LFIA based method can provide broad screening for seven kinds of β-agonist with lowest LODs (0.01 ng·mL^−1^ for CMT). Here, the performance of different types of ELISA and LFIA methods were compared (Table 2), the advantages in terms of broad-screening are obvious: (1) a novel HCR-mAb was applied to develop the broadest screening method for seven kinds of β-agonist in a single test so far. (2) The sensitivity of the FLFIA method is highest and the LOD is as the lowest as 0.01 ng·mL^−1^ (for CMT). (3) The method was validated to be suitable for pork tissue samples. (4) It is fast, easy to operate and low cost.

**Table 2:** Comparison of immunoassays for β-agonists

Compounds	Method	Sample	Analysis of flux	LOD (ng/g)				Reference
CLEN	ELISA	Urine	Single		0.2			[12]
		Liver						
SAL	LFIA	Urine	Single		0.16			[24]
CLEN	LFIA	Urine	Single		0.037			[25]
β-Agonists	Fluorescent LFIA	Urine	Multi-residues	CLEN			0.10	[26]
		Tissue		RAC			0.10	
		Feed		SAL			0.09	
β-Agonists	LFIA	Urine	Multi-residues	CLEN			1.0	[29]
				RAC			1.0	
Brombuterol	Electrochemiluminescence immunosensor	Tissue	Single		3.7×10^−5^			[30]
		Feed						
Brombuterol	LFIA	Tissue	Single		0.38			[31]
		Urine						
β-Agonists	Fluorometric lateral flow immunoassay (FLFIA)	Tissue	Broad screening	CLEN			0.025	This method
				CMT			0.01	
				CBT			0.025	
				MAB			0.025	
				BBT			0.05	
				BCT			0.025	
				BAN			0.05	

### Application of the method

Two contaminated real tissue samples were tested using the method and the results were compared with LC-MS/MS [32]. As listed in Table S3, the semi quantification results of the method are in good agreement with the reference LC-MS/MS method.

## Conclusion

The multi-component FLFIA demonstrated good performances in terms of sensitivity, linearity, reproducibility and accuracy, for simultaneous determination of seven kinds of β-agonist in the real samples. All β-agonists can be quantitatively detected on a single strip within 8 min with a low-cost instrument. The LOD of the 7 β-agonists that was lower than 0.05 ng·mL^−1^ can be achieved. Furthermore, we provided a high performance antibody for more kinds of β-agonist and it can be used to develop high flux and sensitive assay method, such as electrochemical assay etc. Overall, the nanoparticle based strip combined with high performance antibody can be a potential alternative format of the ICA strip for rapid, sensitive and broad spectrum on-site detection of CLEN and its structural analogues as well as for other hazardous substances in screening applications and a variety of other biomedical applications. However, there are so many types of illicit drugs that might exist in animal feed, edible tissue and other sample matrices. Therefore, further study should focus on the development of multi-class assay for interesting hazardous compounds on one strip assay.

## Electronic supplementary material

ESM 1 (DOCX 179 kb)
