# Supplementary material for: A paper-based competitive lateral flow immunoassay for multi β-agonist residues by using a single monoclonal antibody labelled with red fluorescent nanoparticles
Source: Mikrochim Acta. 2018 Feb 22;185(3):191. doi: 10.1007/s00604-018-2730-9 (PMC5823949; doi:10.1007/s00604-018-2730-9)
Supplement: Supplementary file 1 — (DOCX 179 kb) [file 604_2018_2730_MOESM1_ESM.docx]

**Electronic Supporting Material on the Microchimica Acta publication**

**A paper-based competitive lateral flow immunoassay for multi β-agonist residues by using a single monoclonal antibody labelled with red fluorescent nanoparticles**

**Ruiguo Wang, Wei Zhang, Peilong Wang^*,a,b^, Xiaoou Su^*,a,b^**

*a* Key Laboratory of Agro-product Safety and Quality, Ministry of Agriculture, Beijing 100081，P.R. China.

*b* Institute of Quality Standards & Testing Technology for Agro-Products, Chinese Academy of Agricultural Sciences, Beijing 100081, P.R. China.

**Preparation of antibody–FB conjugates**

The HCR-mAb-FB conjugates were prepared by the method described by Wang et al. [26] In brief, HCR-mAb for β-agonists was diluted to 2 mg/mL by using 50mM 2-(N-morpholino) ethanesulfonic acid (pH=5.0). 100 μL of diluted HCR-mAb was mixed with 100μL FB and sonicated for 1 min. The mixture was gently vortexed and settled for 15 min at room temperature. 5 μL of 1-(3-dimethylaminopropyl)-3-ethylcarbodiimide (0.5 g/mL) was spiked into the above mixture and sonicated for 1 min. Proper volume of sodium hydroxide (1 mol/L) was added to adjust the pH to 6.5. The mixture was reacted for 3 h under vortex at room temperature. After that, 20 μL of glycine (1 mol/L) was transferred into above mixture and well-mixed. The resulted HCR-mAb-FB conjugate was rinsed with 0.01 M phosphate buffer (pH=7.4) and dissolved in 100 μL 1% BSA.

**
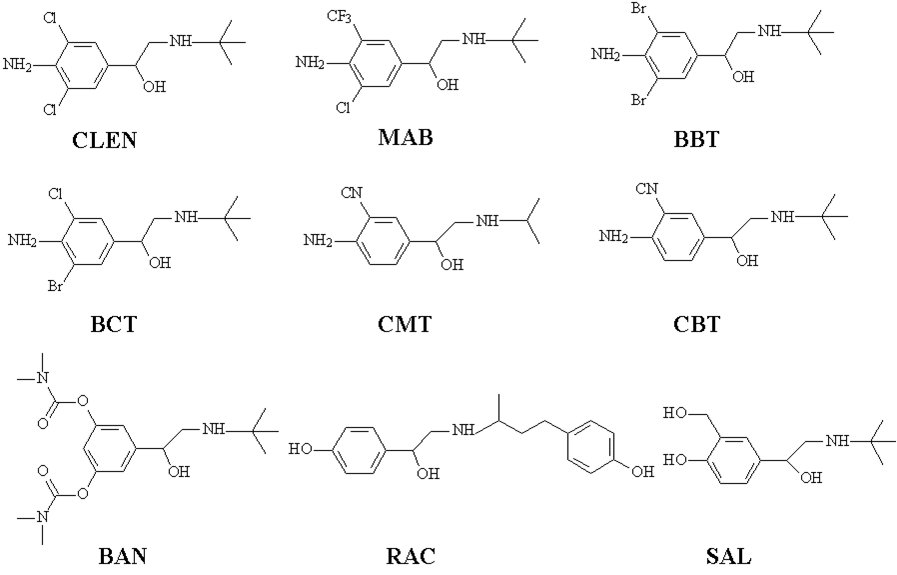
**

**Fig.S1.** Structure of CLEN and it’s structural analogues

**
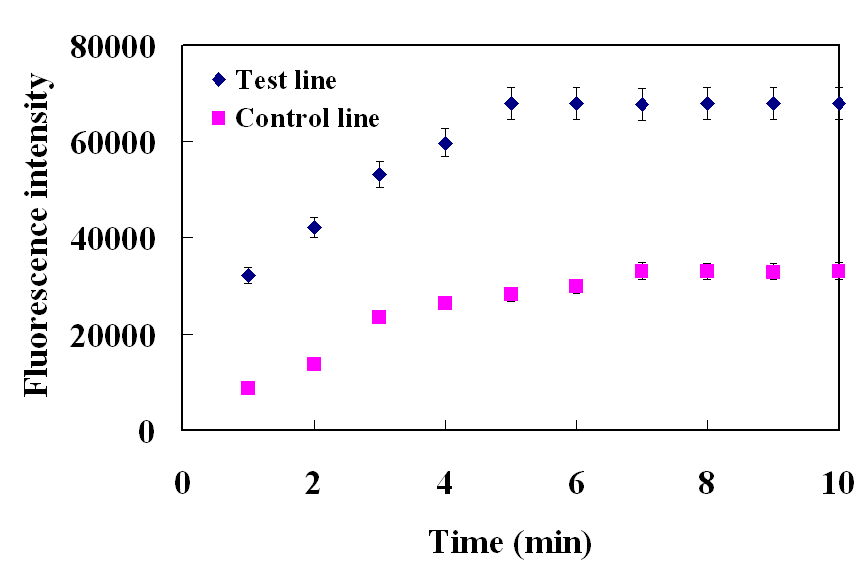
**

**Fig. S2.** Effect of reaction time on fluorescence intensity at the test line and control line on FLFIA strip (Blank swine urine, n=5).

**
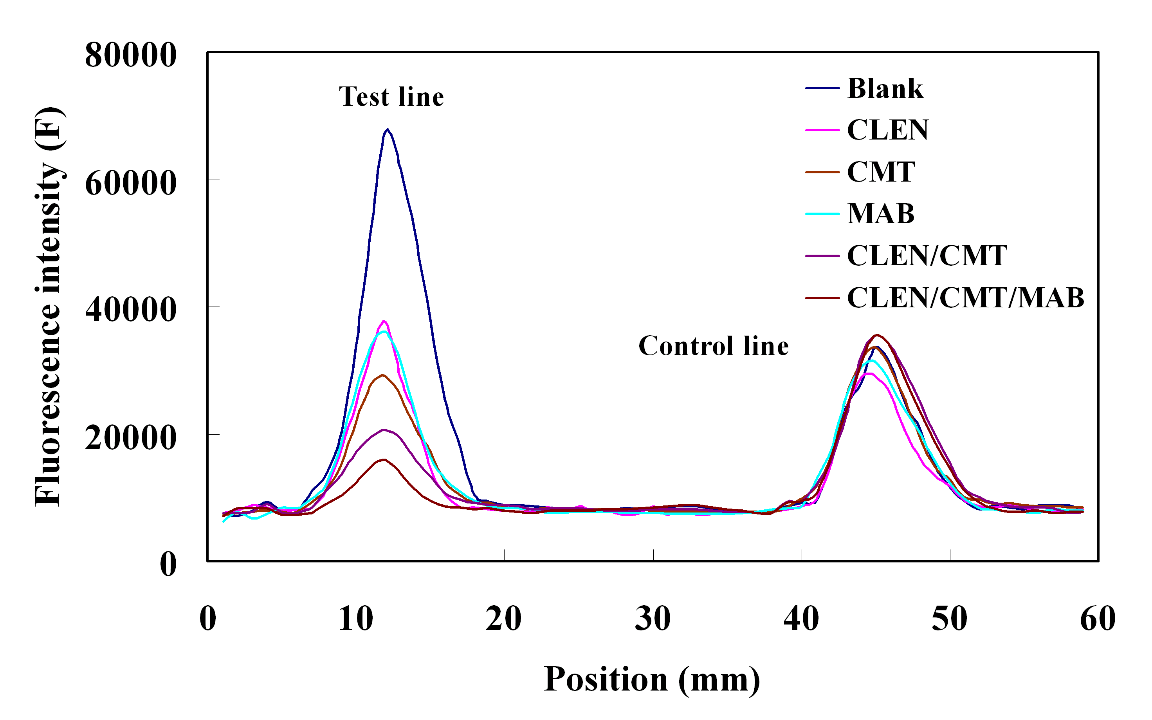
**

**Fig. S3.** Detection of target β-agonists in swine urine in presence of RAC (1.0 ng/mL) and SAL (1.0 ng/mL) using FLFIA method (CLEN 0.1 ng/mL; CMT 0.1 ng/mL; MAB 0.1 ng/mL; CLEN/CMT 0.1/0.1 ng/mL; CLEN/CMT/MAB 0.1/0.1/0.1 ng/mL)

**Table S1**

IC50 values and cross reactivity of HCR-mAb with β-agonists and other competitors.

| Competitors | HCR-mAb of β-agonist | |
| --- | --- | --- |
|  | IC_50_ (ng/mL) | Cross-reactivity (%) |
| CLEN | 0.13 | 100 |
| MAB | 0.1 | 130 |
| BAN | 0.15 | 86.7 |
| BBT | 0.15 | 86.7 |
| CMT | 0.06 | 216.7 |
| CBT | 0.09 | 144.4 |
| BCT | 0.08 | 162.5 |
| RAC | >100 | <0.13 |
| SAL | >100 | <0.13 |
| fenoterol | >100 | <0.13 |
| tulobuterol | >100 | <0.13 |
| epinephrine | >200 | <0.065 |
| dopamine | >500 | <0.026 |
| penicillin | >1000 | <0.013 |
| ampicillin | >1000 | <0.013 |
| kanamycin | >1000 | <0.013 |
| ciprofloxacin | >1000 | <0.013 |

IC50 was the concentrations giving 50% inhibition of HCR-mAb on the test lines of FLFIA. Cross reactivity was expressed as the percentage of IC50 concentrations of the competitors divided.

**Table S2**

Analytical performance of presented broad-screening FLFIA method

| Compounds | Linear range  (ng/mL) | Linear equation | R^2^ | LOD (ng/g) |
| --- | --- | --- | --- | --- |
| CLEN | 0.0~0.8 | y = -5.4574x + 5.3211 | 0.9916 | 0.025 |
| MAB | 0.0~0.8 | y = -5.1535x + 5.2892 | 0.9780 | 0.025 |
| BAN | 0.0~0.8 | y = -5.1215x + 5.4022 | 0.9939 | 0.05 |
| BBT | 0.0~0.8 | y = -3.8182x + 5.2954 | 0.9637 | 0.05 |
| CMT | 0.0~0.8 | y = -5.5661x + 5.1402 | 0.9904 | 0.01 |
| CBT | 0.0~0.8 | y = -5.2201x + 5.1943 | 0.9671 | 0.025 |
| BCT | 0.0~0.8 | y = -5.1450x + 5.2170 | 0.9804 | 0.025 |

**Table S3**

Comparison of the analysis results for the β-agonist in contaminated pork tissue samples by the FLFIA and reference LC-MS/MS. (n=5)

| Sample | Sample 1 | | | | Sample 2 | | | |
| --- | --- | --- | --- | --- | --- | --- | --- | --- |
|  | This method | | LC-MS/MS | | This method | | LC-MS/MS | |
|  | C ^a^ (ng/g) | RSD (%) | C (ng/g) | RSD (%) | C (ng/g) | RSD (%) | C (ng/g) | RSD (%) |
| CLEN | 1.04 | 7.3 | 0.98 | 5.1 | ND | -- | ND | -- |
|  | ND^b^ | --^c^ | ND | -- | ND | -- | ND | -- |
| MAB | ND | -- | ND | -- | ND | -- | ND | -- |
|  | ND | -- | ND | -- | 1.27 | 8.5 | 1.58 | 5.7 |
| BAN | ND | -- | ND | -- | ND | -- | ND | -- |
|  | ND | -- | ND | -- | ND | -- | ND | -- |
| BBT | ND | -- | ND | -- | ND | -- | ND | -- |
|  | ND | -- | ND | -- | ND | -- | ND | -- |
| CMT | ND | -- | ND | -- | ND | -- | ND | -- |
|  | ND | -- | ND | -- | ND | -- | ND | -- |
| CBT | ND | -- | ND | -- | ND | -- | ND | -- |
|  | ND | -- | ND | -- | ND | -- | ND | -- |
| BCT | ND | -- | ND | -- | ND | -- | ND | -- |
|  | ND | -- | ND | -- | ND | -- | ND | -- |

^a^ The concentration of CLEN, RAC and SAL in real samples was detected by using this method and LC-MS/MS.

^b^ No detection of CLEN, RAC and SAL using FLFIA method and LC-MS/MS.

^c^ No RSD.
